# The Role of Peroxisome Proliferator-Activated Receptor and Effects of Its Agonist, Pioglitazone, on a Rat Model of Optic Nerve Crush: PPARγ in Retinal Neuroprotection

**DOI:** 10.1371/journal.pone.0068935

**Published:** 2013-07-18

**Authors:** Juming Zhu, Junfang Zhang, Min Ji, Hongwei Gu, Yue Xu, Chen Chen, Nan Hu

**Affiliations:** Eye Institute, Nantong University, Nantong, China; Universidade Federal do ABC, Brazil

## Abstract

It has been shown that peroxisome proliferators-activated receptor gamma (PPARγ) is beneficial for central nervous system injury. However its role on optic nerve injury remains unknown. In the present study, we examined the change of PPARγ expression in rat retina following optic nerve injury and investigated the effect of pioglitazone (Pio), a PPARγ agonist, on retinal ganglion cells (RGCs) neuroprotection using a rat optic nerve crush (ONC) model. Our results showed that PPARγ mRNA and protein levels were increased after ONC, and most of PPARγ-immunoreactive cells colocalized with Müller cells. Pio treatment significantly enhanced the number of surviving RGCs and inhibited RGCs apoptosis induced by ONC. However, when PPARγ antagonist GW9662 was used, these neuroprotective effects were abolished. In addition, pio attenuated Müller cell activation after ONC. These results indicate that PPARγ appears to protect RGCs from ONC possibly via the reduction of Müller glial activation. It provides evidence that activation of PPARγ may be a potential alternative treatment for RGCs neuroprotection.

## Introduction

Axon injury and loss of retinal ganglion cells (RGCs) are major pathological substrates for permanent visual disability in many ophthalmic diseases, such as glaucoma, optic nerve injury and ischemic optic neuropathy [1], [2]. Several common mechanisms have been hypothesized to underlie the processes of RGCs loss, including neurotrophic factor deprivation [3], glutamate-induced excitotoxicity [4], oxidative stress [5], reactive gliosis [6] and induction of pro-apoptotic pathways [7]. Based on these hypotheses, a variety of studies and strategies for providing neuroprotection to the injured retina have been proposed [8], [9], [10], [11], [12]. However, many studies are still inconclusive and have had low success rates in the transition from the laboratory to human trials. Hence, further research for continued understanding of molecular mechanisms contributing to RGCs death should persist in the hope that this will develop more effective treatments for these diseases.

Peroxisome proliferator-activated receptor-γ (PPARγ) is a ligand - activated transcription factor of nuclear hormone receptor superfamily [13]. It plays a critical role in a variety of biological processes, including adipogenesis, glucose metabolism, angiogenesis and inflammation [14], [15]. Many investigators have focused on the neuroprotective effects of PPARγ against neurological diseases [16]–[21]. Several studies have indicated that PPARγ agonists thiazolidinediones (TZDs, include rosiglitazone, pioglitazone and troglitazone) could prevent or attenuate neurodegeneration in animal models of Alzheimer’s disease [16], Parkinson’s disease [17] and amyotrophic lateral sclerosis [18]. PPARγ agonists also have been shown to provide neuroprotection in acute central nervous system (CNS) insults like cerebral ischemia, spinal cord injury (SCI) and traumatic brain injury (TBI) [19], [20], [21]. Because of the success of PPARγ agonists in multiple models of CNS diseases and their offer of a broad range of potentially protective properties, we hypothesize that PPARγ activation will be beneficial in RGCs protection. In the present study, we investigated the change of PPARγ expression in rat retina following optic nerve crush (ONC) and evaluated the effects of pioglitazone, a United States Food and Drug Administration-approved drug to treat diabetics, on RGCs survival after ONC. In addition, we also observed the relationship between PPARγ and retinal Müller cell activation.

## Materials and Methods

### Animals

One hundred and sixty-two Adult Sprague Dawley rats of both sexes, weighing 200–300 g, were provided by the Experimental Animal Center of Nantong University. All experiments involving animals were carried out in accordance with the US National Institute of Health (NIH) Guide for the Care and Use of Laboratory Animals published by the US National Academy of Sciences and approved by the Administration Committee of Experimental Animals, Jiangsu Province, China.

### Experimental Design

Seventy-two rats were used for PPARγ expression analysis and they were divided into 8 groups (n = 9 at each group): the control (received sham operation), 1 day (d), 3 d, 5 d, 7 d, 14 d, 21 d and 28 d after ONC. In each group, 6 rats were used for RT-PCR and Western blot analysis and 3 rats were used for immunohistochemistry detection.

Ninety rats were enrolled in the pioglitazone (Pio, Takeda Pharmaceutical Co. LTD) treatment experiment and they were randomly divided into 5 groups (n = 18 at each group): 1) sham group: rats were subjected to sham operation and administered vehicle [0.1% dimethyl sulfoxide (DMSO)], 2) vehicle group: rats were subjected to ONC and administered vehicle, 3) Pio group: rats were subjected to ONC and administered Pio (10 mg/kg Pio dissolved in DMSO), 4) GW9662 group: rats were subjected to ONC and administered PPARγ antagonist GW9662 (sigma, 1.5 mg/kg dissolved in DMSO), and 5) Pio+GW9662 group: rats were subjected to ONC and administered Pio+GW9662. Vehicle, Pio or GW9662 were administered by intraperitoneal injection once a day after ONC or sham operation. In each group, 6 rats were used for RGCs Retrograde labeling, 6 rats were used for western blot analysis, and others were used for TUNEL and immunofluorescence observation.

### Optic Nerve Crush

All animals underwent ONC injury or sham operation in the left eye. ONC was performed as previously described with slight modification. Briefly, rats were deeply anesthetized with an intraperitoneal injection of 10% chloral hydrate. An incision was made on the temporal conjunctiva, the lateral rectus muscle was dissected, and the optic nerve was exposed and isolated. Care was taken to avoid damaging the small vessels around the optic nerve. The optic nerve was crushed with a micro- vascular clip for 10 s [22]. Before wound closure the retinal perfusion was checked funduscopically. Animals with severe reduction of the perfusion were excluded. Sham operation was done with the same procedures but without crushing the optic nerve.

### Real-time PCR

To determine the mRNA level of PPARγ in the retina after ON injury, total RNA was extracted from the retina of rats at different time points after ONC using trizol reagent, and cDNA was synthesized from the total RNA using a PrimeScript RT reagent kit (Takara) according to the supplier’s instructions. The reaction in a 25 µL volume containing a 2 µL cDNA template was prepared using SYBR® Premix Ex Taq ™ II (Takara) and run with a Real-Time PCR machine (Applied Biosystems). Primer sequences are as follows: PPARγ: sense, tggagcctaagtttgagtttgc, and antisense, tgaggtctgtcatcttctggag; β-actin: sense, cacccgcgagtacaaccttc, and antisense, cccatacccaccatcacacc (designed and synthesized by the Bioengineering Technology Co., Ltd., Shanghai. China). The PCR conditions were 95°C for 30 s, 95°C for 5 s and 60°C for 34 s for a total of 40 cycles. The mean Ct value for triplicate measurements was used to calculate the expression of target gene with normalization to β-actin according to the 2^−ΔΔCt^ formula.

### Western Blot

Retina tissue were homogenized in a protein lysis buffer (containing 1 M Tris–HCl at pH 7.5, 1% Triton X-100, 1% Nonidet p-40, 10% SDS, 0.5% sodium deoxycholate, 0.5 M EDTA, 10 µg/ml leupeptin,10 µg/ml aprotinin, and 1 mM PMSF) and centrifuged at 12,000×g for 15 min to collect the supernatant. Protein concentrations were estimated by the Bio-Rad protein assay (Bio-Rad Laboratories, Segrate, Milan, Italy). Aliquots of tissue lysates containing an equal amount of protein were resolved by 10% SDS-PAGE and transferred to PVDF membranes. The membranes were blocked with 5% nonfat dry milk and allowed to incubate with primary antibodies against PPARγ (Santa Cruz; 1∶100), Bax (Santa Cruz; 1∶200), Bcl-2 (Santa Cruz; 1∶200) and GADPH (Santa Cruz; 1∶1000) at 4°C overnight. After reaction with second antibodies HRP-conjugated goat anti-rabbit IgG (Sigma; 1∶2000) at 37°C for 1 h, immunoreactivity was detected by enhanced chemiluminescence kit (Amersham Pharmacia Biotech, Piscataway, NJ, USA) and visualized by autoradiography.

### Sections and Immunohistochemistry

After rats were anesthetized and perfused, eyeballs were removed and postfixed for 24 h in 4% paraformaldehyde, and then dehydrated by 30% sucrose. The tissues were embedded in optimum cutting temperature (OCT) compound and sectioned (10 µm thick) with a cryostat (CM1900, Leica, Bensheim, Germany). Endogenous peroxidase was blocked with 0.3% H_2_O_2_ and nonspecific binding sites were blocked with a solution containing 2% donkey serum, 2% bovine serum albumin and 0.2% TritonX-100. Then, the sections were incubated with the antibody against PPARγ (Santa Cruz; 1∶50) at 4°C overnight. Immunoreactions were visualized using the polink-2 plus polymer HRP detection system for rabbit primary antibodies (GBI Co., Ltd.). The slides were counterstained with hematoxylin and examined under a light microscope (Leica, Germany).

### Retrograde Labeling and Counting of RGCs

To assess for the effect of Pio on RGCs survival, retrograde labeling of RGCs was performed 1 week before ONC according to our previously described methods. After anesthetization, the rats were placed in the stereotactic apparatus (Stoelting, Wood Dale, IL, USA) and the brain surface was exposed by perforating the parietal bone to facilitate dye injection. 2 µL of 2% FluoroGold (FG; Biotium, Hayward, CA, USA) was injected into both superior colliculi and dorsal lateral geniculate nuclei, and the skin was sutured. 7 days after ONC, rats from different treatment groups were sacrificed and eyeballs were enucleated and placed in 4% paraformaldehyde for 6–8 h. The whole retina was then carefully dissected, flattened and mounted with the vitreous side up on slides. Photographs were captured using a fluorescent microscope (Leica, Germany) and FG- labeled RGCs were counted in a masked fashion by the same investigator using automated particle counting software in ImagePro Version 6.0 (Media Cybernetics, Bethesda, USA). The number of labeled cells in 12 photographs of each retina (three photographs per retinal quadrant) at 1/6, 3/6, and 5/6 of the retinal radius were summed together and expressed as mean RGC densities/mm^2^ for each group.

### TUNEL Staining

TUNEL staining was performed using a fluorescein in situ cell death detection kit (Roche Applied Science, Germany) according to the manufacturer’s instructions. The nuclei of the cells were stained with hochest (Santa Cruz; 1∶2000) at room temperature for 15 min. The double-stained cells were observed with fluorescence microscope (Leica, Germany). The number of TUNEL positive cells in the ganglion cell layer (GCL) was counted in a masked fashion using automated particle counting software in ImagePro Version 6.0. A minimum of four sections (50 µm apart) per animal were used and three separate GCL regions were examined for each section.

### Immunofluorescence

The retinal sections were incubated for 2 h in blocking solution and then overnight at 4°C with primary antibodies rabbit anti-PPARγ (Santa Cruz ) at 1∶50, mouse anti- neuronal nuclei (NeuN; Santa Cruz) at 1∶100, mouse anti- glutamine synthetase (GS; Santa Cruz) at 1∶100, and mouse anti-glial fibrillary acidic protein (GFAP; Sigma) at 1∶400. Further reaction with CY3-conjugated secondary antibody donkey anti-mouse IgG at 1∶200 and FITC- conjugated secondary antibody donkey anti-rabbit IgG at 1∶200 (all Jackson ImmunoResearch Laboratories Inc., West Grove, PA) were applied at 4°C for 2 h. Sections were then rinsed, counterstained with hochest for 5 min, and observed under a Leica fluorescence microscope.

### Statistical Analysis

All data were expressed as mean ± SD. Differences among groups were analyzed by one-way ANOVA, followed by Tukey’s post hoc multiple comparison tests. All statistical analyses were carried out by the aid of STATA 7.0 software package (Stata Corp., College Station, TX), and significance levels were set at p<0.05.

## Results

### The Expression of PPARγ in Retina after ONC

**Figure 1:**
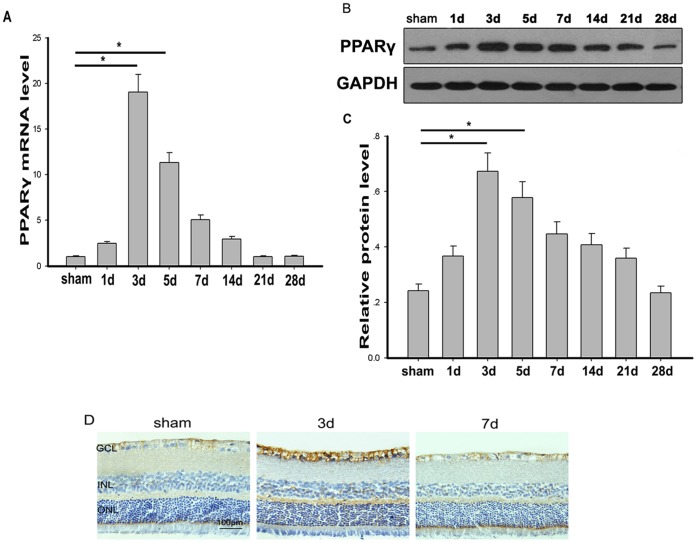
The expression of PPARγ in rat retina after ONC. A. The expression of PPARγ mRNA was increased after ONC. The highest expression was found on day 3, then returned to basal values on day 14 and remained unchanged until the end of the observation period of 28 days(n = 6 in each group). *p<0.05. B. Western blot analysis of PPARγ protein in the retina. C. The expression of PPARγ protein relative to GAPDH was determined by measuring the optical density using ImageJ software(n = 6 in each group). *p<0.05. D. Immunohistochemical staining of PPARγ in rat retina. High PPARγ immunoreactivity was observed in the nerve fiber layer (NFL), ganglion cell layer (GCL), inner neuclear layer (INL) and outer plexiform layer (OPL) 3 days after ONC. Bar = 100 µm.

The expression of PPARγ in retina in both control and experimental groups was examined by real-time PCR at each time point after ONC. As the results shown in Fig. 1A, we found a low level of PPARγ mRNA in non-crushed retina, however, the PPARγ gene expression significantly increased after optic nerve injury, peaking on day 3 following ONC and returning to normal level on day 14. These PCR results were in line with the western blot analyses showing that the amount of PPARγ protein was significantly higher after ONC (Fig. 1 B&C). The expression of PPARγ protein was further investigated by immunohistochemistry (Fig. 1D). In control group, PPARγ positive cells were distributed mainly in the inner retinal layers, and also observed in a scattered pattern in inner nuclear layer (INL). Three days after ONC, PPARγ expression was dramatically increased, mainly in the nerve fiber layer (NFL), ganglion cell layer (GCL), INL and outer plexiform layer (OPL). PPARγ immunoreactivity was decreased at 7 days after ONC.

**Figure 2:**
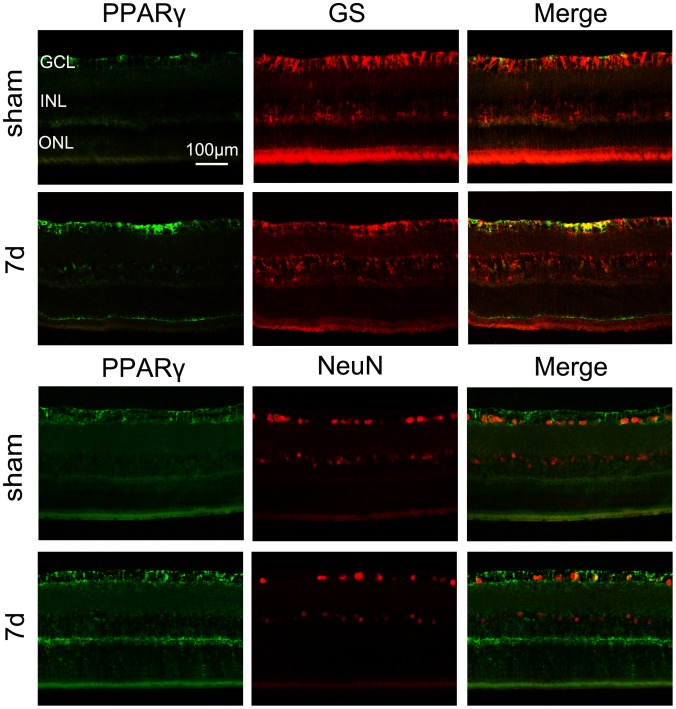
Double immunoﬂuorescence staining for PPARγ (green)/GS (red) and PPARγ (green)/NeuN (red) on the normal retina and the retina 7 days after ONC. Most of PPARγ positive cells are colocalized with GS positive Müller cells, not within NeuN positive RGCs. Bar = 100 µm.

To identify cell subsets in the inner retinal layers that express PPARγ, rat retinal sections were coimmunostained to detect PPARγ and either GS (staining for Müller cells) or Neun (staining for RGCs). As shown in Fig. 2, most of PPARγ positive cells were colocalized with GS positive Müller cells, especially at their end-feet. However, only parts of PPARγ immunoreactivity overlapped with Neun positive RGCs.

### Pio Protects RGCs from Injury-induced Apoptosis

**Figure 3:**
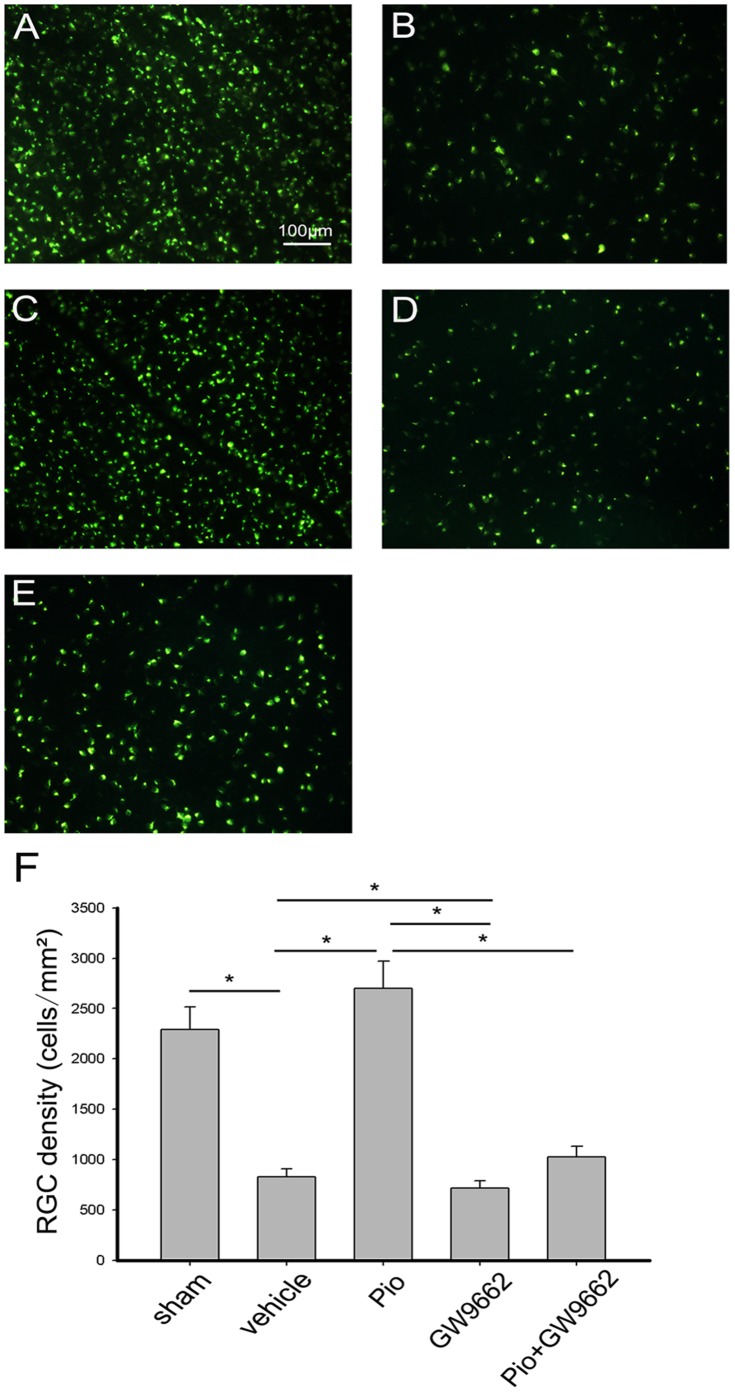
Effect of pioglitazone (Pio) on RGCs survival after optic nerve crush (ONC). (A) A representative photograph of RGCs labeled with FluoroGold (FG) injected into the superior colliculus in normal retina (sham group). (B–E) A representative photograph of FG-labeled RGCs, 7 days after ONC with intraperitoneal injection of DMSO(B), Pio(C), GW9662(D) and Pio+ GW9662(E). Bar = 100 µm. (F) Quantitative analysis of the number of FG-labeled RGCs 7 days after ONC. Data represent the means ± SD (n = 6 in each group). *p<0.05.

The number of survival RGCs was determined by FG retrograde labeling. The mean density of RGCs in the uninjured retina (sham group) was 2290/mm^2^. Seven days after ONC, RGCs density decreased to 826/mm^2^ in the retina of vehicle treated rats. In the Pio group, the number of FG labeled cells was 2700/mm^2^ (Fig. 3). The results demonstrate that RGCs survival rate increased by approximately 30.6% in the Pio-treated group as compared to the vehicle group. To examine whether the neuroprotection afforded by Pio was PPARγ-dependent, specific PPARγ- antagonist GW9662 was used together with Pio. As expected, the neuroprotective effect was abrogated in the presence of GW9662 (Fig. 3). When GW9662 was used alone, RGCs density was 717/mm^2^, significantly lower than those in vehicle group (Fig. 3).

**Figure 4:**
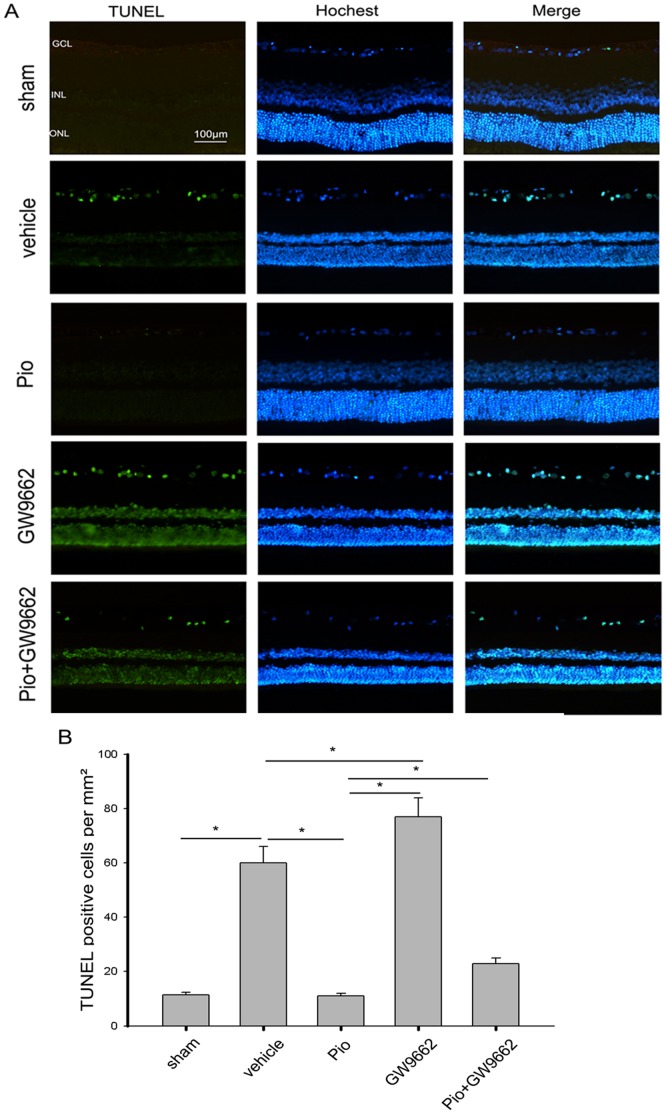
The anti-apoptotic effect of Pio on RGCs after ONC. (A) Representative of the terminal dUTP nick end labeling (TUNEL) staining in retinal sections of each group at 7 days after ONC. Hochest dye was used for nuclear staining. (B) TUNEL-positive cells in the RGCs layer at 7 days after ONC(n = 6 in each group). *p<0.05. Bar = 100 µm.

**Figure 5:**
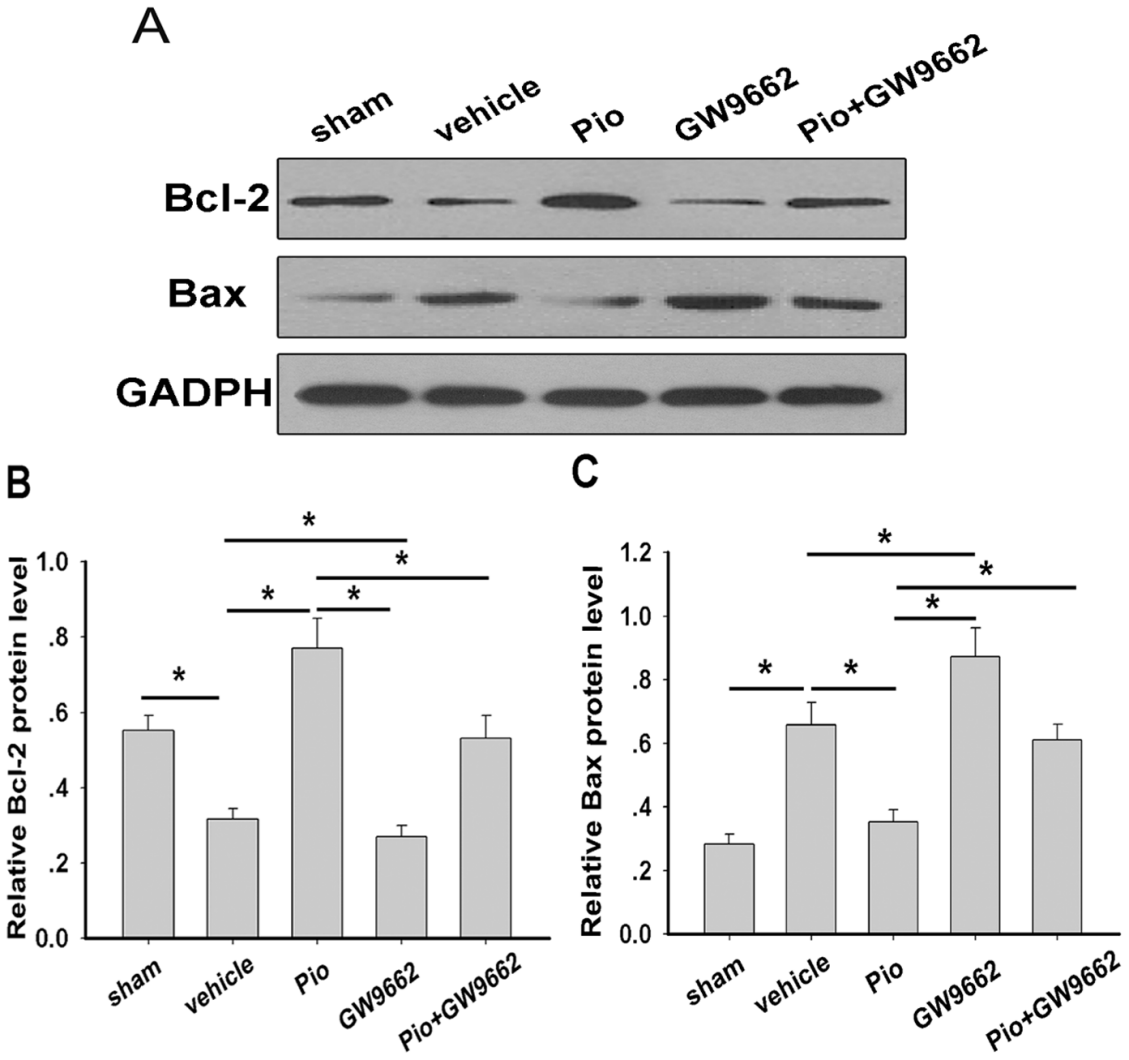
Effect of Pio on anti- and pro- apoptotic protein expression in rat retina at 7 days after ONC. Western blot analysis indicated that Pio treatment could increase the expression of Bcl-2 (A and B), and decrease the expression of Bax (A and C). Bars represent the means ± SD (n = 6 in each group). *p<0.05.

The neuroprotective effects of Pio were further examined by TUNEL staining. The result showed that about 11cells/mm^2^ underwent apoptosis in the vehicle group (control), while 60/mm^2^ in the sham group underwent apoptosis. However, there were significantly fewer apoptotic cells (12/mm^2^) in the Pio group. Again, the inhibition of apoptosis was blocked by administration of GW9662 (24/mm^2^ in Pio+GW9662 group). When GW9662 was used alone, the number of the apoptotic cells was approximately 78/mm^2^, significantly higher than those in vehicle group (Fig. 4). In addition, western blot was used to determine anti-and pro-apoptotic proteins. Compared with vehicle group, Pio significantly increased Bcl-2 protein levels but reduced Bax protein levels in the retina, indicating reduced apoptosis. When GW9662 was used alone, Bax protein levels were increased but Bcl-2 protein levels were reduced. When GW9662 was used together with Pio, no Bax or Bcl-2 alterations were observed (Fig. 5).

### Pio Attenuates Müller Cell Activation

**Figure 6:**
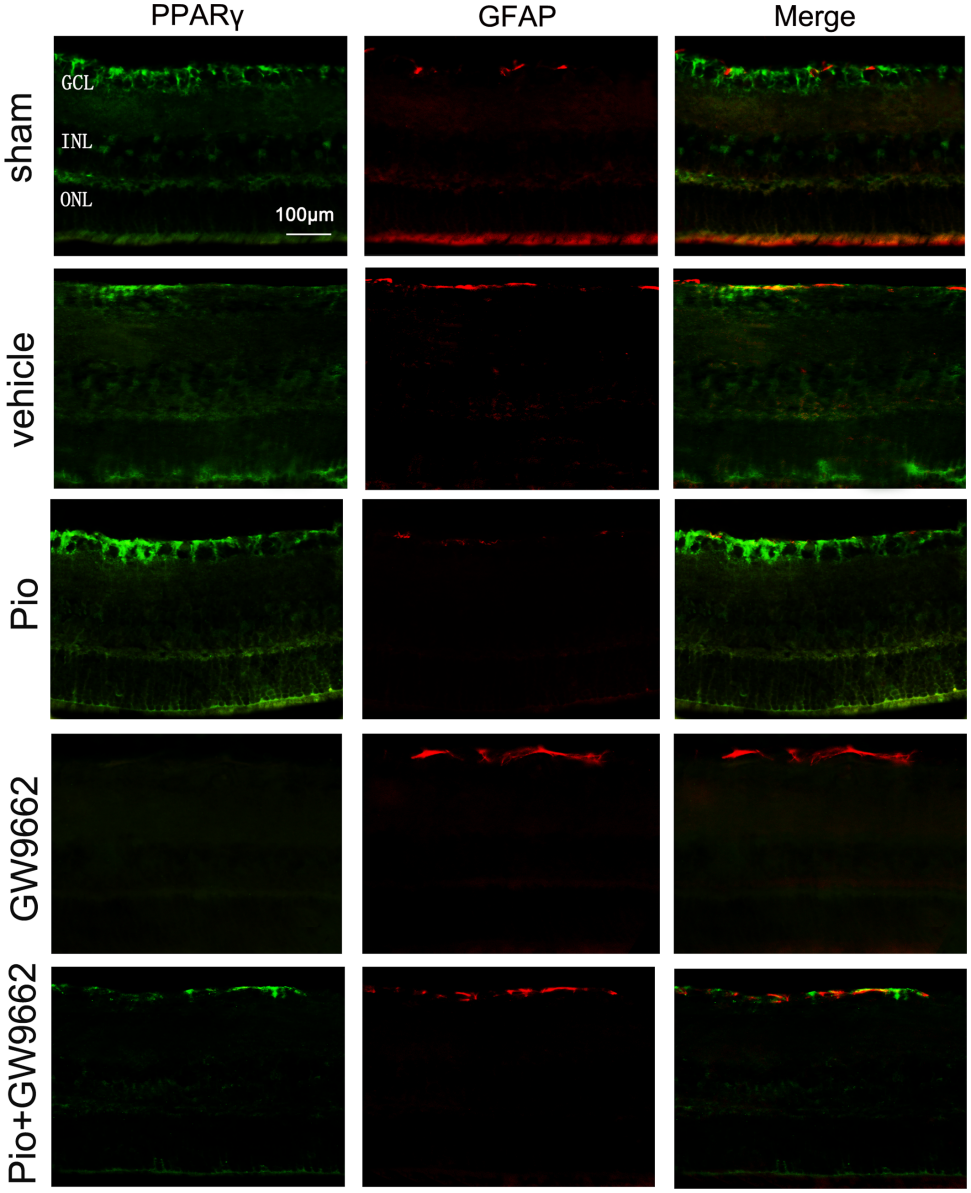
Effect of Pio on Müller cell activation in retina. Immunoreactivity for PPARγ (green) and GFAP (red) in rat retinal sections of each group at 7days after ONC. Bar = 100 µm.

Increased expression of GFAP was observed in the retina of vehicle group at 7days after ONC (Fig. 6). In the Pio group, GFAP expression was significantly decreased, accompanied by increased PPARγ immunoreactivity. These changes were blocked when Pio and GW9662 were administered simultaneously. When GW9662 was given alone, the intensity of GFAP staining was dramatically increased but no PPARγ immunoreactivity was detected.

## Discussion

In the present study, we examined changes in the expression of PPARγ in injured retina and investigated the role of PPARγ agonist Pio on RGCs survival. We chose an ONC rat model, since ONC rodent model has been widely used in studying RGCs degeneration and the pathophysiology of glaucoma as well as other optic neuropathies [23], [24], [25], [26]. There are two main findings of this work. The first is that PPARγ is up-regulated in rat retina after ONC. The second is that PPARγ agonist Pio protects RGCs from ONC-induced neuronal apoptosis possibly via the reduction of glial activation. To our knowledge, this is the first report characterizing the expression of PPARγ in retina under pathologic conditions associated with optic nerve injury. Furthermore, it is the first report to show the effects of Pio on RGCs protection after ONC.

Previous studies have indicated that PPARγ is heterogeneously expressed in the mammalian eye [27], [28], [29], [30]. It was found to be most prominent in the retinal pigmented epithelium, neuroretina, choriocapillaris, choroidal endothelial cells, corneal epithelium and endothelium. However, there are no studies on the changes in the PPARγ immunoreactivity and mRNA/protein levels in the retina after optic nerve injury. In the present study, we found the expression of PPARγ was up-regulated by ONC. PPARγ mRNA and protein levels increased starting from 1 day, were highest at 3 day, and then gradually decreased with time. The results suggest that endogenous PPARγ plays an intrinsic role in the progression of traumatic optic neuropathies. In addition, our immunoﬂuorescence staining demonstrated that most of PPARγ- immunoreactive cells colocalized with Müller cells, suggesting the relationship between PPARγ and Müller glial cells. PPARγ expression has been reported to be up-regulated in the ischemic brain [21], [31] and following traumatic brain injury [32], and PPARγ immunoreactivity is mostly localized in the microglia and astrocytes [33]. Our present results are supported by these previous studies.

In recent years, experimental studies in models of cerebral ischemia/reperfusion injury, ischemic stroke, intracerebral hemorrhage, traumatic brain injury and spinal cord injury have revealed a crucial role of PPARγ and its ligands in attenuating neuronal cell death in the injured CNS [19], [20], [21], [31], [32]. However, to our knowledge, only one previous study has reported the effect of PPARγ on RGCs [34]. Their results showed that two PPARγ ligands, 15-deoxy-D(12,14)-prostaglandin J2 and troglitazone, protected RGC-5, an established transformed rat retinal ganglion cell line, against glutamate cytotoxicity. In the present study, we demonstrated the neuroprotective effects of PPARγ on RGCs in vivo. It was observed at 7 days post injury that animals treated with Pio exhibited an increased RGCs survival and decreased RGCs apoptosis compared to animals treated with either vehicle or Pio and the PPARγ antagonist GW9662 together. The PPARγ antagonist was able to block the protective effects of Pio, indicating that activation of the PPARγ pathway is critical to attenuating RGCs loss after ONC. The results provided the rationale for pharmacotherapeutic targeting of PPARγ for RGCs protection. Interestingly, animals treated with PPARγ antagonist GW9662 showed significantly greater RGCs loss and apoptosis compared to vehicle group. This evidence further supported the theory that PPARγ activation by its endogenous ligands is an essential neuroprotective event after ONC. This neuroprotection can be enhanced by providing exogenous PPARγ agonists. Similar results have been reported in transient focal ischemia [35] and traumatic brain injury [32].

Since our results indicated that most of PPARγ expression was localized in Müller glial cells, we further examined the expression of GFAP, a marker for Müller cell activation in retina. In virtually all models studied, retinal Müller cells increase the expression of GFAP in response to neuronal degenerative processes in the retina [36]. As expected, retinal GFAP expression increased in optic nerve injured eyes as compared to eyes that received sham operation. GW9662 induced an additional significant increase in the expression of GFAP whereas Pio significantly decreased the GFAP expression, indicating PPARγ acted as a negative regulator of Müller cell activation in the ONC-damaged retina. The neuroprotective mechanisms associated with PPARγ agonists have not yet been fully identified. It has been reported that the neuroprotective effects of PPARγ agonists are associated with down-regulation of the inflammatory response [19], [21], [32]. We didn’t examine the inflammatory factors in this study, but our results showed Pio inhibited the Müller cell activation. In the retina, Müller cell is a predominant glial cell that maintains the microenvironment and supports RGC function [36]. Under stress, they become activated and produce pro-inflammatory cytokines and their involvement in immune and inflammatory responses may exert detrimental effects [37], [38], [39]. Thus, we thought the neuroprotective effect of Pio on RGCs at least partly depended on attenuating reactive gliosis and reducing inflammation.

In conclusion, our study showed that ONC led to increased expression of PPARγ in the retina and treatment with PPARγ agonist Pio induces significant neuronal survival and protection from apoptotic cell death. Our results suggest that the activation of PPARγ may be a potential alternative treatment for RGCs neuroprotection. Future studies will be required to understand the mechanisms of PPARγ-mediated neuroprotection in retina.
